# Optimization of *Aspergillus niger* rock phosphate solubilization in solid-state fermentation and use of the resulting product as a P fertilizer

**DOI:** 10.1111/1751-7915.12289

**Published:** 2015-06-25

**Authors:** Gilberto de Oliveira Mendes, Nina Morena Rêgo Muniz da Silva, Thalita Cardoso Anastácio, Nikolay Bojkov Vassilev, José Ivo Ribeiro, Ivo Ribeiro da Silva, Maurício Dutra Costa

**Affiliations:** 1 Departamento de Microbiologia, Universidade Federal de ViçosaViçosa, Brazil; 2 Instituto de Ciências Agrárias, Universidade Federal de UberlândiaMonte Carmelo, Brazil; 3 Departamento de Ingeniería Química, Universidad de GranadaGranada, Spain; 4 Departamento de Estatística and, Universidade Federal de ViçosaViçosa, Brazil; 5 Departamento de Solos, Universidade Federal de ViçosaViçosa, Brazil; 6 Pesquisador Bolsista do Conselho Nacional de Desenvolvimento Científico e Tecnológico (CNPq)Brasília, Brazil

## Abstract

A biotechnological strategy for the production of an alternative P fertilizer is described in this work. The fertilizer was produced through rock phosphate (RP) solubilization by *Aspergillus niger* in a solid-state fermentation (SSF) with sugarcane bagasse as substrate. SSF conditions were optimized by the surface response methodology after an initial screening of factors with significant effect on RP solubilization. The optimized levels of the factors were 865 mg of biochar, 250 mg of RP, 270 mg of sucrose and 6.2 ml of water per gram of bagasse. At this optimal setting, 8.6 mg of water-soluble P per gram of bagasse was achieved, representing an increase of 2.4 times over the non-optimized condition. The optimized SSF product was partially incinerated at 350°C (SB-350) and 500°C (SB-500) to reduce its volume and, consequently, increase P concentration. The post-processed formulations of the SSF product were evaluated in a soil–plant experiment. The formulations SB-350 and SB-500 increased the growth and P uptake of common bean plants (*Phaseolus vulgaris* L.) when compared with the non-treated RP. Furthermore, these two formulations had a yield relative to triple superphosphate of 60% (on a dry mass basis). Besides increasing P concentration, incineration improved the SSF product performance probably by decreasing microbial immobilization of nutrients during the decomposition of the remaining SSF substrate. The process proposed is a promising alternative for the management of P fertilization since it enables the utilization of low-solubility RPs and relies on the use of inexpensive materials.

## Introduction

Phosphorus (P) is one of the most limiting elements for crop production in most soils. The volume of use of P fertilizers in the world is second only to nitrogen fertilizers (Food and Agriculture Organization of the United Nations, faostat.fao.org). The main natural P reserve is composed by apatite [Ca_5_(PO_4_)_3_(F,Cl,OH)] rock phosphates (RPs), from which soluble fertilizers are obtained mainly by treatment with strong acids. This process requires high energy input, besides causing an almost complete dissolution of the ore, which results in the release of undesirable contaminants into gas streams, byproduct streams, and into phosphate products (Goldstein *et al*., 1993). Furthermore, the RP reserves are a finite resource, and there is a great concern about their impending depletion, with predictions of scarcity for the next few centuries (Cordell and White, 2011; Neset and Cordell, 2012).

The use of phosphate-solubilizing microorganisms (PSMs) is an attractive alternative for the management of P in agriculture. PSMs can mobilize insoluble P through the production of organic acids, which can be exchanged with phosphate in insoluble compounds (Bolan *et al*., 1994; Kpomblekou-A and Tabatabai, 1994), and, to a lesser extent, by the release of H^+^ during their growth (Illmer and Schinner, 1995). PSMs can be applied directly to the soil together with poorly soluble P sources (Jain *et al*., 2010), such as RP, or used in *in vitro* systems to produce high-solubility P fertilizers from RP (Vassilev *et al*., 2014). The latter option allows the control of the variables that govern microbial RP solubilization, something difficult to attain in the soil. In such *in vitro* systems, the fungus *A. niger*, known by its high ability to produce organic acids, has been widely applied for RP solubilization in submerged or solid-state fermentations with a range of substrates (Vassilev *et al*., 1997; 1998; 2014; Mendes *et al*., 2013a).

An interesting strategy proposed for RP solubilization is the use of agro-industrial wastes as substrate for microbial growth in solid-state fermentation (SSF) (Vassilev and Vassileva, 2003; Vassilev *et al*., 2014). Generally, SSFs give higher product yields than submerged fermentations, probably because the SSF conditions are closer to the natural habitat of microorganisms (Pandey, 2003). Recently, our group obtained an *A. niger* isolate (FS1) able to solubilize RP in a SSF using sugarcane bagasse as substrate (Mendes *et al*., 2013a). In that work, *A. niger* FS1 solubilized up to 3.6 mg of P per gram of sugarcane bagasse. Optimized conditions were not established, although the data showed that P solubilization could be improved by increasing RP doses. Furthermore, recent findings showed that fungal P solubilization is inhibited by fluoride released from RP (Mendes *et al*., 2013b). The addition of biochar to the fermentation medium alleviates fluoride toxicity and increases the production of organic acids, consequently increasing P solubilization (Mendes *et al*., 2014b). Thus, it is reasonable to assume that this SSF-based RP solubilization system could be significantly improved by the optimization of the levels of RP and biochar.

The optimization of process parameters is a crucial step for the development of bioprocesses in SSF (Pandey, 2003). Vassilev and colleagues (2014) recommend that at least the inoculum concentration, moisture, temperature and pH should be optimized. The incubation time should also be considered for RP solubilization systems, since the concentration of soluble P fluctuates during microbial growth (Vassilev *et al*., 1996; Mendes *et al*., 2013b). The medium composition is also important. The Czapek's solution has been usually adopted in SSF-based RP solubilization systems (Vassilev *et al*., 1996; 2006; Vassileva *et al*., 1998; Oliveira, 2011; Mendes *et al*., 2013a). However, recently our group evaluated separately the effect of the nitrogen and carbon sources of this solution in an SSF with sugarcane bagasse. It was demonstrated that the addition of nitrogen had no effect on P solubilization by *A. niger* (Oliveira, 2011; Mendes *et al*., 2013a). Conversely, an organic carbon source must be added if soluble sugars are not readily available in the solid substrate (Oliveira, 2011). Nevertheless, there is no report about the necessity of the addition of other minerals present in the Czapek's solution. Another interesting factor to explore is the addition of methanol. There are various works reporting increased production of citric acid following the addition of methanol to the fermentation medium (Hang and Woodams, 1998; Roukas, 1999; 2000). Thus, by increasing the production of citric acid, the addition of methanol could improve P solubilization.

The product of SSF-based RP solubilization systems have been applied to soil as an alternative P fertilizer, increasing plant growth and P uptake (Vassilev *et al*., 1996; 2006; Vassilev and Vassileva, 2003). However, some agro-industrial wastes are difficult to mineralize in SSF, which can result in a product with low P content. Even though a high quantity of P is solubilized, if little mineralization occurs, the final volume of solid material will be high and, consequently, the P concentration low. Thus, high quantities of the SSF product would be necessary to supply the P requirement of plants, which is not advantageous in a practical context. Thus, the main objectives of this work were to optimize the SSF conditions that allow maximum RP solubilization by *A. niger* FS1 and to develop a post-processing step aiming at increasing the P concentration of the SSF product through its partial incineration.

## Results

### Optimization of RP solubilization

Among the ten factors screened, only five presented a significant effect on P solubilization by *A. niger* FS1 in the SSF (Table 1). At the range evaluated, increases in the doses of minerals, RP, biochar, and sucrose resulted in a positive effect on P solubilization, while those in moisture content had a negative effect. For the subsequent experiments, the non-significant factors were fixed at the following values: initial pH, unadjusted (4.6); temperature, 30°C; incubation time, 7 days; inoculum size, ∼ 10^6^ conidia flask^−1^; and methanol, not added.

**Table 1:** Screening of factors affecting the solubilization of Araxá rock phosphate by *Aspergillus niger* FS1 in a solid-state fermentation with sugarcane bagasse as substrate

Factor	Low level (−1)	High level (+1)	Effect^a^
Sucrose (mg g^−1^)^b^	25	50	0.686*
Minerals^c^(ml g^−1^)	0	1	0.104*
Initial pH	Unadjusted (4.6)	7.0	0.044
Moisture (ml g^−1^)	5.7	8	−0.360*
Temperature (°C)	25	30	−0.054
Incubation time (days)	7	13	0.014
Inoculum size (conidia flask^−1^)	10^5^	10^7^	−0.030
Rock phosphate (mg g^−1^)	15	45	0.244*
Methanol (ml g^−1^)	0	0.05	−0.022
Biochar (mg g^−1^)	0	100	0.390*

*Significant by the *t* test (*P* < 0.05).

a *Effect = m_+_ – m_–_* (*m_+_* and *m_−_* are the mean of solubilized P for the high and low level of each factor, respectively).

b Values are expressed per gram of dry sugarcane bagasse added at the beginning.

c A solution of minerals was used (adapted from Czapek's medium) (g l^−1^): MgSO_4_.7H_2_O, 3; KCl, 3; FeCl_3_.6H_2_O, 0.108.

The significant factors were gradually varied according to the steepest ascent technique and a near-stationary region was apparently achieved, reaching about 5 mg of soluble P per gram of bagasse (Supporting Information, Table S.4). The factor levels obtained by the steepest ascent technique were used as the central point in an experiment under composite central design (CCD) to fit a response surface (Supporting Information, Table S.5, Exp. VI). However, the regression analysis showed that the linear component of the response surface was significant whereas the quadratic was not (data not shown), indicating that the factor levels should be increased even more. Thus, new points were added to the model in sequential experiments (Supporting Information, Table S.5, Exp. VII-IX) until a maximum of solubilized P was reached. These experiments showed that the level of some factors was far from the optimal, especially that of biochar, which was increased from 136 to 850 mg g^−1^ of bagasse (Supporting Information, Table S.5). Conversely, the data indicated that the addition of minerals was unnecessary. By gathering all the experimental data obtained in the experiments VI-IX in a sole model, a maximum of 9.27 mg P g^−1^ was predicted to be solubilized. However, in a confirmatory assay using these levels, only 7.59 mg P g^−1^ was achieved, indicating that the model adjustment needed improvements.

**Figure 1:**
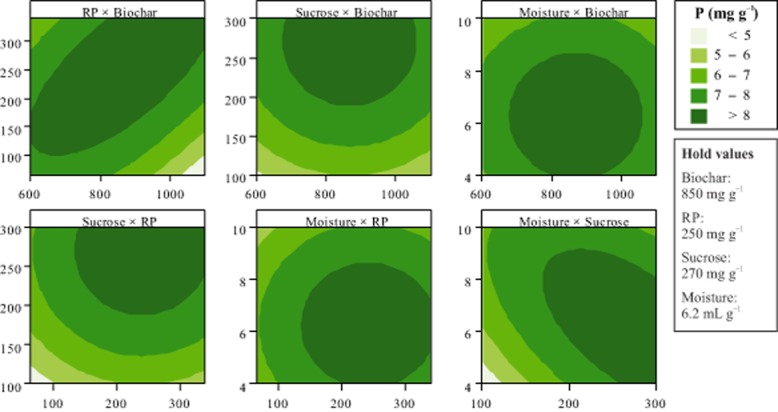
Levels of P solubilized by *Aspergillus niger* FS1 in a SSF in response to different combinations of biochar, RP, sucrose and moisture. The fermentations were performed for 7 days at 30°C using sugarcane bagasse as substrate. In each panel, the variables not presented were hold at the optimum value as indicated in the box.

A new experiment under CCD was set up using the likely optimized factor levels obtained in the first attempt as the central point of the model (Table 2). The regression analysis showed that only the quadratic coefficient of RP was not statistically significant (data not shown). Thus, an additional point (Table 2, Exp. XI) was added to the model to improve the estimate of the quadratic coefficient for RP. The second-order model fitted (using data in uncoded units) with only significant coefficients (*P* < 0.05) was:

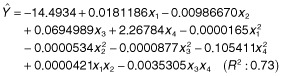
where *Y* is the predicted value of solubilized P (mg g^−1^ of bagasse), *x_1_* is the level of biochar (mg g^−1^ of bagasse), *x_2_* is the level of RP (mg g^−1^ of bagasse), *x_3_* is the level of sucrose (mg g^−1^ of bagasse), and *x_4_* is the level of moisture (ml g^−1^ of bagasse). The maximum solubilized P predicted using the equation (8.6 mg g^−1^) was obtained with 865 mg of biochar, 250 mg of RP, 270 mg of sucrose and 6.2 ml of water per gram of sugarcane bagasse. By plotting the equation in a contour plot, the optimal region can be seen with a concentration of solubilized P higher than 8 mg g^−1^ (Fig. 1). Two points within this region (the maximum predicted and a combination using less RP) were assayed to confirm the model adjustment. For both combinations, the observed data were in good agreement with those predicted (Table 2).

**Table 2:** Solubilized phosphorus by *Aspergillus niger* FS1 in a solid-state fermentation as a function of combinations of different levels of biochar, rock phosphate (RP), sucrose, and moisture

Experiment	Biochar	RP	Sucrose	Moisture	Solubilized P (mg g^−1^)	
	(mg g^−1^)^a^	(mg g^−1^)	(mg g^−1^)	(ml g^−1^)	Measured	Predicted^b^
X	725 (−1)^c^	120 (−1)	150 (−1)	5.5 (−1)	6.48	6.50
	975 (1)	120	150	5.5	5.51	5.28
	725	230 (1)	150	5.5	7.25	6.72
	975	230	150	5.5	6.65	6.66
	725	120	250 (1)	5.5	8.23	8.01
	975	120	250	5.5	6.76	6.79
	725	230	250	5.5	8.09	8.22
	975	230	250	5.5	8.64	8.16
	725	120	150	8.5 (1)	7.59	7.29
	975	120	150	8.5	6.19	6.07
	725	230	150	8.5	8.68	7.51
	975	230	150	8.5	7.76	7.45
	725	120	250	8.5	7.78	7.73
	975	120	250	8.5	6.95	6.51
	725	230	250	8.5	8.08	7.95
	975	230	250	8.5	9.01	7.89
	850 (0)	175 (0)	200 (0)	7 (0)	7.92	8.05
	600 (−2)	175	200	7	8.40	7.66
	1100 (2)	175	200	7	6.16	6.38
	850	65 (−2)	200	7	6.27	6.60
	850	285 (2)	200	7	7.96	8.20
	850	175	100 (−2)	7	5.77	6.20
	850	175	300 (2)	7	7.50	8.14
	850	175	200	4 (−2)	6.75	6.84
	850	175	200	10 (2)	7.06	7.36
XI Additional point to improve the estimate of the square coefficient for RP	850	340	200	7	7.58	7.79
Confirmatory points	865	250	270	6.2	9.43	8.60
	750	150	270	6.2	8.77	8.34

The experiment was set up under a composite central design. The fermentations were performed for 7 days at 30°C using sugarcane bagasse as substrate.

a Values are expressed per gram of dry sugarcane bagasse added at the beginning.

b Calculated with the fitted regression equation: 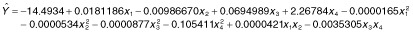 (Y, predicted solubilized P; x_1_, level of biochar; x_2_, level of RP; x_3_, level of sucrose; x_4_, level of moisture).

c Values within the parentheses are the coded levels.

### Soil–plant experiment

The SSF product obtained after the optimization was evaluated as to its efficiency in supplying P to plants when applied to the soil. Because of the low proportion of P in the SSF product obtained, the effect of incinerating the product was evaluated as an alternative to increase the P concentration. The incineration at 350°C and 500°C caused a loss of mass of 64% and 77%, respectively. Consequently, the concentration of total P in the formulations SB-350 and SB-500 increased to 4.4% and 7.4% respectively (Table 3). Conversely, the fraction of water-soluble P in the formulations SB-350 and SB-500 was lower than that of the SB-70. The pH of the formulations SB-70, SB-350 and SB-500 was 3.0, 7.6, and 9.5, respectively.

**Table 3:** Phosphorus content (grams P per 100 g of fertilizer) of the fertilizers used in the soil–plant experiment

Fertilizer	Total P^a^	H_2_O-soluble P^b^	CA-soluble P^c^	NAC-soluble P^d^
SB-70	1.7	0.5	0.9	0.4
SB-350	4.4	0.3	0.4	0.4
SB-500	7.4	0.1	3.5	1.3
TSP	22.3	18.0	19.6	20.5
ARP	14.0	0.3	1.7	0.4

a Extracted with 6:1 nitric-hydrochloric acid mixture.

b Soluble in H_2_O (1:200).

c Soluble in 2% citric acid (1:100).

d Soluble in neutral ammonium citrate.

ARP, Araxá rock phosphate; SB-70, SSF product dried at 70°C; SB-350, SSF product incinerated at 350°C; SB-500, SSF product incinerated at 500°C; TSP, triple superphosphate.

The growth of the bean plants fertilized with the formulations SB-350 and SB-500 was superior to that obtained with the SB-70 (Table 4). SB-70 had no significant effect on plant growth when compared with the fertilization treatment with RP and with the non-amended soil. On average, the formulations SB-350 and SB-500 presented a yield relative to triple superphosphate (TSP) of 60% (dry mass basis). The P content of plants that received the three formulations of the SSF product was superior to that observed in the non-amended soil. Also, the total P uptake by the plants fertilized with the formulations was higher than that of the non-amended soil. The treatment fertilized with the formulation SB-70 presented the lowest value of total N uptake among the formulations.

**Table 4:** Effect of different phosphate fertilizers on the growth and phosphorus and nitrogen uptake by bean plants (*Phaseolus vulgaris*) cultivated for five weeks

Fertilizer	Dry matter (g pot−1)	Yield relative to TSP (%)	P content (mg g−1)	Total P uptake (mg pot−1)	Total N uptake (mg pot−1)
SB-70	2.00^c^ ± 0.08	32^b^	1.80^b^ ± 0.34	3.62^cd^ ± 0.76	38.4^c^ ± 8.5
SB-350	3.62^b^ ± 0.50	58^a^	1.30^c^ ± 0.14	4.67^bc^ ± 0.44	73.2^ab^ ± 10.1
SB-500	3.82^b^ ± 0.31	61^a^	1.53^bc^ ± 0.09	5.83^b^ ± 0.65	82.6^ab^ ± 7.5
TSP	6.26^a^ ± 0.76	100	2.88^a^ ± 0.22	17.86^a^ ± 0.81	93.9^a^ ± 20.3
ARP	2.25^c^ ± 1.01	36^b^	1.23^cd^ ± 0.05	2.74^de^ ± 1.18	60.4^bc^ ± 10.3
Control	1.52^c^ ± 0.22	24^b^	0.88^d^ ± 0.05	1.33^e^ ± 0.23	58.2^bc^ ± 9.7

Values are means of four replicates ± standard deviation. Means sharing a letter are not significantly different (Tukey test, *P* < 0.05).

The superscript letters refer to the statistical test described in this footnote.

ARP, Araxá rock phosphate; Control, non-amended soil; SB-70, SSF product dried at 70°C; SB-350, SSF product incinerated at 350°C; SB-500, SSF product incinerated at 500°C; TSP, triple superphosphate.

## Discussion

A 2.4-fold increase in RP solubilization by *A. niger* was achieved after optimization of the parameters of an SSF using sugarcane bagasse as substrate. An initial screening revealed that the quantity of P solubilized by *A. niger* is affected by the level of biochar, RP, sucrose and water added to the medium. By applying the sequential methodology proposed by Box and Wilson (1951), a maximum of 8.6 mg P g^−1^ was reached with the combination of 865 mg of biochar, 250 mg of RP, 270 mg of sucrose and 6.2 ml of water per gram of sugarcane bagasse (Fig. 1). The region of maximum solubilization highlighted in Fig. 1 (solubilized P > 8 mg g^−1^) is composed by a range of combinations among the factors. This allows the exploration of combinations with reduced cost and equivalent soluble P in the final product. One combination using less RP was assayed to prove this and, as predicted by the regression equation, a solubilization higher than 8 mg P g^−1^ of bagasse was measured (Table 2). The optimized solubilization reached was significantly higher than that previously obtained in this system (3.6 mg g^−1^) (Mendes *et al*., 2013a). The system proposed here is also remarkably superior to other solubilization systems based on SSF or submerged fermentations, with increases above 300% (Table 5). Works dealing with the optimization of microbial RP systems are still rare and the results obtained herein point out that there is a great potential to be explored.

**Table 5:** Comparison of the yield of different microbial RP solubilization systems with the present work

System and substrate	Soluble P (mg g^−1^)^a^	References	Increase obtained in the present work
Solid-state fermentation			
Sugar-beet waste	2.9	(Vassilev *et al*., 1996)	297%
Olive-cake	0.5	(Vassilev *et al*., 2006)	1720%
Sugarcane bagasse	3.6	(Mendes *et al*., 2013a)	239%
Submerged fermentation			
Vinasse	1.2	(Nahas *et al*., 1990)	717%
Olive mill wastewater	0.58	(Vassilev *et al*., 1997)	1483%
Glucose (NBRIP^b^medium)	0.1	(Xiao *et al*., 2008)	8600%
Starch (Pykovskaya's broth)	1.6	(Ahuja and D'Souza, 2008)	538%

a When not stated in this unit, values were converted using initial substrate mass for SSF and considering 1 ml equal to 1 g for submerged fermentations.

b National Botanical Research Institute's phosphate growth medium.

The factors submitted to optimization were screened from a group of variables previously described as affecting P solubilization (Vassilev *et al*., 1996; Oliveira, 2011; Mendes *et al*., 2013a, b) or fungal metabolism (Vassilev *et al*., 2014), especially the pathways for the production of organic acids (Hang and Woodams, 1998; Roukas, 1999; 2000; Magnuson and Lasure, 2004). The addition of a nitrogen source was not evaluated because previous works of our group with this same solubilization system demonstrated that the nitrogen content of the bagasse was enough to cover the fungal requirement (Oliveira, 2011; Mendes *et al*., 2013a). Regarding the carbon source, sucrose and glucose were equally efficient for this solubilization system (Oliveira, 2011), but sucrose was chosen because it is cheaper and easier to obtain in local markets. Nevertheless, a variety of carbohydrate substrates has been applied in fermentations (Vassilev *et al*., 2014) and could be evaluated as alternative carbon source according to local availability. From the cultivation parameters, only the factors related to the medium composition (addition of sucrose and minerals and moisture content) showed significant effect on RP solubilization. The dose of RP and the addition of biochar to the fermentation medium also presented a significant effect on the level of P solubilized by *A. niger*. The positive effect of biochar on RP solubilization is related with its ability to adsorb toxic compounds released from RP, namely fluoride (Mendes *et al*., 2013b), and to increase the production of organic acids (Mendes *et al*., 2014b). After the optimization, the product obtained was submitted to a post-processing to increase the P concentration by the reduction in its volume through partial incineration. As expected, the proportion of P in the SSF products incinerated increased (Table 3). However, the water-soluble fraction decreased. After the incineration, the alkalinity of the SSF product increased, which probably caused a reprecipitation of P with metallic cations, mainly Ca^2+^ (Illmer and Schinner, 1995). Nevertheless, pure synthetic apatites and poorly soluble P salts, such as Ca_3_(PO_4_)_2_, are more soluble than the natural apatite found in RPs (Schneider *et al*., 2010; Mendes *et al*., 2014a). Thus, it is expected that under soil conditions, especially in acid soils or acidic microsites, part of the reprecipitated P can be released faster than it would be from the untreated RP. This hypothesis is supported by the data of total P uptake by bean plants (Table 4). Plants fertilized with SB-500, which had the lowest water-soluble P fraction (0.1% P), accumulated a higher quantity of P than that fertilized with SB-70. Furthermore, the formulation SB-500 presented the highest fractions of CA- and NAC-soluble P among the SSF product formulations (Table 3), showing that part of the P is in a readily soluble form. The formulations SB-350 and SB-500 were equally efficient at supplying P and promoting plant growth (Table 4). Based on dry matter, these formulations presented an average yield relative to the TSP of 60%. Furthermore, the formulations were superior to the non-treated RP. These results are very interesting because a significant improvement of Araxá RP yield was achieved using a simple biotechnological strategy. Further studies are necessary to determine the agronomic effectiveness of the SSF product formulations. Another interesting topic for studies is the long-term effect of these SSF product formulations, since part of the P is in a less soluble form that could be released slowly along the time. Apparently, the amount of P which was taken up by the plants was not the main factor limiting the growth of plants fertilized with the different formulations, since SB-70 and SB-350 resulted in similar P uptake while the growth of plants fertilized with the formulation SB-70 was significantly lower (Table 4). The plants fertilized with the formulation SB-70 presented some chlorotic leaves (data not shown) and the lowest accumulation of N among the formulations (Table 4). Since sugarcane bagasse possesses a high C/N ratio (Magalhães *et al*., 2006), it is likely that microbial immobilization of N took place during its decomposition (Craine *et al*., 2007; Manzoni *et al*., 2008), which could explain the reduced plant growth. Thus, besides reducing the SSF product volume, the incineration also contributes to decrease the possibility of microbial nutrient immobilization. A two-step biotechnological process for the production of a P-rich SSF product is proposed in this work. In the first step, soluble P was obtained from RP through fungal solubilization in an optimized SSF based on inexpensive materials, such as sugarcane bagasse, biochar, RP and sucrose. The second step consisted in incinerating the SSF product to reduce the volume and, consequently, increase the P concentration. The incinerated product was applied to the soil and increased the growth and P uptake by common bean plants. The procedure of incineration used in this work needed external input of energy. Since both SSF products incinerated at 350°C and 500°C had the same effect on plant growth and P uptake, the lowest temperature should be recommended due to the lower energy requirement. Another economic alternative is to burn the SSF product in boilers to produce power and use the ash as a P-rich fertilizer, as is already done with the sugarcane bagasse in the industry (Stanmore, 2010).

## Conclusions

An increase of 2.4 times in RP solubilization by *A. niger* in an SSF with sugarcane bagasse as substrate was achieved after optimization. The factors controlling the concentration of solubilized P in the final product are the amount of biochar, sucrose, RP, and water. The optimized product was applied to the soil after partial incineration at 350°C or 500°C and increased the growth and P uptake by plants. These results are of great interest for the management of P fertilization given that the process is based on the use of inexpensive materials that can contribute to lower the costs of the final product.

## Experimental procedures

### Microorganism and fermentation conditions

The strain *A. niger* FS1 was obtained from the Collection of Phosphate-Solubilizing Fungi, Institute of Biotechnology Applied to Agriculture (BIOAGRO), Universidade Federal de Viçosa, Viçosa, MG, Brazil. The fungus was maintained at 30°C on petri dishes containing potato dextrose agar. The fungal inoculum was prepared by collecting conidia from cultures up to 7 days old in a 0.1% (v/v) Tween 80 solution. SSFs were conducted in 250 ml Erlenmeyer flasks with 5 g of dry sugarcane bagasse. In total, 11 experiments were performed to optimize RP solubilization. Unless otherwise stated, flasks were inoculated with 1 ml of a conidial suspension with ∼ 10^6^ conidia ml^−1^ and incubated at 30°C for 7 days. The other factors were added to the successive optimization experiments at the concentrations presented in the tables described in the section ‘Optimization of RP solubilization’ below.

### Rock phosphate, sugarcane bagasse and biochar

The RP from Araxá (Brazil) used as the insoluble P source is a fluorapatite [Ca_5_(PO_4_)_3_F] with 13.97% of P and particle size < 75 μm in diameter (Mendes *et al*., 2013b). The sugarcane bagasse was obtained from a sugar-alcohol mill (Jatiboca, Urucânia, Brazil). Before use, the bagasse was dried and ground into fragments of up to 2 mm. The bagasse contained 0.75% of soluble sugars and its elemental composition was (mg kg^−1^): N, 2800; P, 500; K, 500; Ca, 400; Mg, 600; S, 700; Zn, 11; Fe, 907; Mn, 35; Cu, 2; and B, 9.2 (Oliveira, 2011). The biochar was produced by pyrolysis of biomass wastes of holm oak (*Quercus ilex*) at 480°C and supplied by Piroeco Bioenergy (Málaga, Spain). The biochar had a particle size less than 2 mm, a fixed carbon content of 85.56%, a content of volatiles of 12.24%, an ash content of 2.2% and a pH of 8.7. Its elemental composition (mg kg^−1^) was: N, 5000; P, 2400; K, 11700; Mg, 5600; Mn, 781; Zn, 22; Cu, 13; Ni, 11; Pb, 1.4; Cr, 0.7; Cd, 0.07. Soluble P content in 2% citric acid (1:100) was 899.9 mg P per kilogram of biochar (Mendes *et al*., 2014b).

### Optimization of RP solubilization

#### Screening

Ten factors with potential effect on RP solubilization by *A. niger* were screened in two 2^6-1^ fractional factorial experiments (one-half fraction) (Barros Neto *et al*., 2010). Each factor was evaluated at two levels coded as −1 (low level) and 1 (high level) in the experimental design (Table 1). In the first screening, the effect of the addition of biochar, initial pH, temperature, inoculum size, methanol, and the addition of a mineral solution [adapted from Czapek's medium (g l^−1^): MgSO_4_.7H_2_O, 3; KCl, 3; FeCl_3_.6H_2_O, 0.108] was studied. The combinations of these factors (coded levels) in the experimental design are shown in Supporting Information, Table S.1. The factors with statistically significant effect in the first screening were selected and pooled with new factors, namely RP, sucrose, moisture content, and incubation time, for the second screening. The combinations are shown in Supporting Information, Table S.2. The main effect of each factor and the two-way interaction effect were calculated according to the equations (Almeida *et al*., 2013):

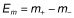


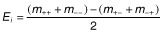
where *E_m_* is the main effect, *E_i_* is the two-way interaction effect, *m_+_* is the mean of solubilized P (P_sol_) for the high level of one factor, *m_−_* is the mean of P_sol_ for the low level of one factor, *m_++_* is the mean of P_sol_ of the high level of two factors, *m_−−_* is the mean of P_sol_ of the low level of two factors, *m_+−_* is the mean of P_sol_ of the high level of factor 1 and low level of factor 2, and *m_−+_* is the mean of P_sol_ of the low level of factor 1 and high level of factor 2. The effects were submitted to analysis of variance (ANOVA) to check if they were different from zero at 5% probability. The factors with significant effect were selected for optimization. In the ANOVA, the third-, fourth-, fifth- and sixth-order interaction effects were used as the residual effect. The experimental design and all the calculations and analyses were done using the option DOE (Design of Experiments) of the statistical software Minitab 16.1.

#### Steepest ascent technique

Initially, a 2^5-2^ fractional factorial experiment (one-fourth fraction) with three replications at the central point (coded as 0) was performed to determine the derivative coefficients (variation in the level of solubilized P as a function of the variation in the level of the factor) of each screened factor (Supporting Information, Table S.3). The data of solubilized P measured (dependent variable) were used to fit a linear polynomial model by multiple linear regression from which the derivative coefficients were obtained. The experimental design and analyses were done using the option DOE of Minitab. After the attainment of the derivative coefficients, the calculation of the path in direction of the expected maximum P solubilization was done as described by Box and Wilson (1951) (Supporting Information, Table S.4). The changes in the level of the factors were calculated based on the change in the level of the factor with the highest product between the variation unit and the coefficient (Unit × *f_t_*). Two sequential experiments were performed in order to reach a near-stationary region, i.e. the maximal response (solubilized P) obtained by proportional increases of the factor levels, which is expected to be near of the theoretical maximum of the process (Box and Wilson, 1951).

#### Surface response

The factor levels at the near-stationary region were used as the central point (coded as 0) in a CCD (Barros Neto *et al*., 2010). Two points above the central point (coded as 1 and 2) and two points below (coded as −1 and −2) completed the experimental design. Two attempts to fit the response surface were performed and the combinations of factors in the experimental designs are shown in the Table 2 and Table S.5 (Supporting Information). The data of solubilized P obtained were used to fit a quadratic polynomial model by a multiple regression procedure using the least squares method. The largest model adopted was (Almeida *et al*., 2013):

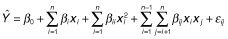
where *Y* is the predicted concentration of solubilized P, *i* and *j* assume a value from 1 to the total number of factors (n), *β_0_* is the intercept term, *β_i_* is the linear coefficient, *β_ii_* is the coefficient of the quadratic term, *β_ij_* is the interaction coefficient, *x_i_* and *x_j_* are the levels of the factors, and *ε_ij_* is the experimental error. An ANOVA with a lack-of-fit test for the response surface was performed, and the coefficients were tested using the *t* test up to 5% probability. The non-significant coefficients were removed from the model. The coefficient of determination (R^2^) was considered to check the quality of the adjustment of the model. The experimental design and all the calculations and analyses were done using the option DOE of Minitab.

### Soil–plant experiment

The optimized SSF product was evaluated in a soil–plant trial under three different formulations obtained from the following post-processing: drying at 70°C (SB-70), incineration in a muffle at 350°C for 4 h (SB-350) and incineration in a muffle at 500°C for 4 h (SB-500). The temperatures of incineration were defined in a preliminary test aiming at identifying different rates of loss of mass (data not shown). The SSF product formulations were applied to pots containing 1 kg of a sandy-clay soil, whose characteristics are presented in Table 6. The high-solubility TSP fertilizer was used as the positive control and the Araxá RP as the negative control. A control without any P fertilizer was also included. The P content of the fertilizers is shown in Table 3. All the fertilizers were applied at a quantity equivalent to 300 mg P pot^−1^ (based on total P content) and homogenized with the whole soil mass (Novais *et al*., 1991). In order to guarantee that P was the only limiting nutrient, the other macro and micronutrients were applied to the soil before the application of the P fertilizers according to recommendations for greenhouse experiments (Novais *et al*., 1991).

**Table 6:** Physical and chemical properties of the soil used in the soil–plant experiment

Property	Value
pH (H_2_O)	5.70
Cation-Exchange Capacity at pH 7 (cmol_c_ kg^−1^)	8.21
Phosphorus (mg kg^−1^)^a^	5.6
Potassium (mg kg^−1^)^a^	112
Calcium (cmol_c_ kg^−1^)^b^	2.11
Magnesium (cmol_c_ kg^−1^)^b^	1.31
Iron (mg kg^−1^)^a^	108.5
Zinc (mg kg^−1^)^a^	1.68
Copper (mg kg^−1^)^a^	1.33
Manganese (mg kg^−1^)^a^	37.6
Aluminum (cmol_c_ kg^−1^)^b^	0.1
Organic matter (g kg^−1^)^c^	41.6
Sand (%)	54
Silt (%)	4
Clay (%)	42
Textural class	Sandy clay

a Extracted with Mehlich I.

b Extracted with KCl 1 mol l^−1^.

c Walkley–Black method.

Five seeds of common bean (*Phaseolus vulgaris* L.) were sown in each pot. After 5 days, the seedlings were thinned to two per pot. The plants were grown in a greenhouse under natural light during 5 weeks. The pots received distilled water daily to maintain moisture at about 70% of the water holding capacity. The experiment contained six treatments (five P sources and a non-amended soil as control) and was organized under a completely randomized design with four replications. The data were submitted to ANOVA and the means of the treatments compared using the Tukey test (*P* < 0.05).

### Analytical methods

Soluble P in the SSF products was extracted by adding 100 ml of distilled water to each flask and shaking at 150 r.p.m. for 1 h. The extract was filtered through a quantitative filter paper (P free, 15 to 17 μm pore size) and soluble P was determined spectrophotometrically using the vanadate-molybdate reagent (Fluka Cat. No 94685) (Clesceri *et al*., 1999). The content of solubilized P was expressed based on the initial mass of dried sugarcane bagasse. After cultivation, the plants were harvested and the shoot dry mass recorded after drying at 70°C until constant weight. The yield related to TSP was expressed as the percentage of mass accumulated in a specific treatment in relation to that accumulated in the TSP control. The dried and ground plant tissue was digested using a 3:1 nitric-perchloric acid mixture (Miller, 1998) and the P content was determined spectrophotometrically using the method of the phosphomolybdate complex (Braga and Defelipo, 1974). N content was determined using the Kjeldahl method (Bremner, 1960).

## Supporting Information

**Table S1.** Combinations of factors in a 2^6-1^ fractional factorial design (one-half fraction) and corresponding P solubilized from Araxá rock phosphate by *Aspergillus niger* FS1 in a solid-state fermentation with sugarcane bagasse as substrate. Each factor was studied at two levels, coded as −1 (low level) and 1 (high level), which are shown in Table 1. **Table S2.** Combinations of factors in a 2^6-1^ fractional factorial design (one-half fraction) and corresponding P solubilized from Araxá rock phosphate by *Aspergillus niger* FS1 in a solid-state fermentation with sugarcane bagasse as substrate. Each factor was studied at two levels, coded as −1 (low level) and 1 (high level), which are shown in Table 1. **Table S3.** Solubilized phosphorus by *Aspergillus niger* FS1 in a solid-state fermentation as a function of combinations of different levels of biochar, rock phosphate (RP), sucrose, minerals, and moisture. The combinations were set up according to a 2^5-2^ fractional factorial design (one-fourth fraction) and the fermentations were performed for 7 days at 30°C using sugarcane bagasse as substrate. **Table S4.** Steepest ascent technique used to achieve a near-stationary region. The data used in the path calculation were obtained in the experiment III (Table S3). The changes in the factor levels were based on a 5- and 10-unit change in sucrose (highest ‘Unit x *f_t_*’ value) in the experiments IV and V, respectively. **Table S5.** Solubilized phosphorus by *Aspergillus niger* FS1 in a solid-state fermentation as a function of combinations of different levels of biochar, rock phosphate (RP), sucrose, minerals, and moisture. Experiment VI was performed under a composite central design. Additional points were included in the design (experiments VII–IX) in order to improve the model adjustment. The fermentations were performed for 7 days at 30°C using sugarcane bagasse as substrate.
