# Supplementary material for: Optimization of Aspergillus niger rock phosphate solubilization in solid-state fermentation and use of the resulting product as a P fertilizer
Source: Microb Biotechnol. 2015 Jun 25;8(6):930–9. doi: 10.1111/1751-7915.12289 (PMC4621446; doi:10.1111/1751-7915.12289)
Supplement: Supplementary file 1 — Table S1. Combinations of factors in a 26-1 fractional factorial design (one-half fraction) and corresponding P solubilized from Araxá rock phosphate by Aspergillus niger FS1 in a solid-state fermentation with sugarcane bagasse as substrate. Each factor was studied at two levels, coded as −1 (low level) and 1 (high level), which are shown in Table 1. Table S2. Combinations of factors in a 26-1 fractional factorial design (one-half fraction) and corresponding P solubilized from Araxá rock phosphate by Aspergillus niger FS1 in a solid-state fermentation with sugarcane bagasse as substrate. Each factor was studied at two levels, coded as −1 (low level) and 1 (high level), which are shown in Table 1. Table S3. Solubilized phosphorus by Aspergillus niger FS1 in a solid-state fermentation as a function of combinations of different levels of biochar, rock phosphate (RP), sucrose, minerals, and moisture. The combinations were set up according to a 25-2 fractional factorial design (one-fourth fraction) and the fermentations were performed for 7 days at 30°C using sugarcane bagasse as substrate. Table S4. Steepest ascent technique used to achieve a near-stationary region. The data used in the path calculation were obtained in the experiment III (Table S3). The changes in the factor levels were based on a 5- and 10-unit change in sucrose (highest ‘Unit x ft’ value) in the experiments IV and V, respectively. Table S5. Solubilized phosphorus by Aspergillus niger FS1 in a solid-state fermentation as a function of combinations of different levels of biochar, rock phosphate (RP), sucrose, minerals, and moisture. Experiment VI was performed under a composite central design. Additional points were included in the design (experiments VII–IX) in order to improve the model adjustment. The fermentations were performed for 7 days at 30°C using sugarcane bagasse as substrate. [file mbt20008-0930-sd1.docx]

**Table S1.** Combinations of factors in a 2^6-1^ fractional factorial design^a^ (½ fraction) and corresponding P solubilized from Araxá rock phosphate by *Aspergillus niger* FS1 in a solid-state fermentation with sugarcane bagasse as substrate. Each factor was studied at two levels, coded as -1 (low level) and 1 (high level), which are shown in Table 1.

| Biochar | pH | Temperature | Inoculum size | Methanol | Minerals | Solubilized P (mg g^-1^)^b^ |
| --- | --- | --- | --- | --- | --- | --- |
| -1 | -1 | -1 | -1 | -1 | -1 | 2.11 |
| 1 | -1 | -1 | -1 | -1 | 1 | 2.34 |
| -1 | 1 | -1 | -1 | -1 | 1 | 2.14 |
| 1 | 1 | -1 | -1 | -1 | -1 | 2.32 |
| -1 | -1 | 1 | -1 | -1 | 1 | 2.21 |
| 1 | -1 | 1 | -1 | -1 | -1 | 2.42 |
| -1 | 1 | 1 | -1 | -1 | -1 | 1.94 |
| 1 | 1 | 1 | -1 | -1 | 1 | 2.68 |
| -1 | -1 | -1 | 1 | -1 | 1 | 2.21 |
| 1 | -1 | -1 | 1 | -1 | -1 | 2.49 |
| -1 | 1 | -1 | 1 | -1 | -1 | 1.93 |
| 1 | 1 | -1 | 1 | -1 | 1 | 2.59 |
| -1 | -1 | 1 | 1 | -1 | -1 | 1.76 |
| 1 | -1 | 1 | 1 | -1 | 1 | 2.32 |
| -1 | 1 | 1 | 1 | -1 | 1 | 2.05 |
| 1 | 1 | 1 | 1 | -1 | -1 | 2.30 |
| -1 | -1 | -1 | -1 | 1 | 1 | 1.99 |
| 1 | -1 | -1 | -1 | 1 | -1 | 2.54 |
| -1 | 1 | -1 | -1 | 1 | -1 | 1.98 |
| 1 | 1 | -1 | -1 | 1 | 1 | 2.46 |
| -1 | -1 | 1 | -1 | 1 | -1 | 2.09 |
| 1 | -1 | 1 | -1 | 1 | 1 | 2.18 |
| -1 | 1 | 1 | -1 | 1 | 1 | 2.22 |
| 1 | 1 | 1 | -1 | 1 | -1 | 2.26 |
| -1 | -1 | -1 | 1 | 1 | -1 | 1.93 |
| 1 | -1 | -1 | 1 | 1 | 1 | 2.47 |
| -1 | 1 | -1 | 1 | 1 | 1 | 2.08 |
| 1 | 1 | -1 | 1 | 1 | -1 | 2.51 |
| -1 | -1 | 1 | 1 | 1 | 1 | 1.94 |
| 1 | -1 | 1 | 1 | 1 | -1 | 2.29 |
| -1 | 1 | 1 | 1 | 1 | -1 | 1.96 |
| 1 | 1 | 1 | 1 | 1 | 1 | 2.58 |

^a^ Experiment I.

^b^ Values are expressed per gram of dry sugarcane bagasse added at the beginning.

**Table S2.** Combinations of factors in a 2^6-1^ fractional factorial design^a^ (½ fraction) and corresponding P solubilized from Araxá rock phosphate by *Aspergillus niger* FS1 in a solid-state fermentation with sugarcane bagasse as substrate. Each factor was studied at two levels, coded as -1 (low level) and 1 (high level), which are shown in Table 1.

| Biochar | Sucrose | Rock phosphate | Moisture | Incubation time | Minerals | Solubilized P (mg g^-1^)^b^ |
| --- | --- | --- | --- | --- | --- | --- |
| -1 | -1 | -1 | -1 | -1 | -1 | 2.52 |
| 1 | -1 | -1 | -1 | -1 | 1 | 2.71 |
| -1 | 1 | -1 | -1 | -1 | 1 | 3.10 |
| 1 | 1 | -1 | -1 | -1 | -1 | 3.29 |
| -1 | -1 | 1 | -1 | -1 | 1 | 2.64 |
| 1 | -1 | 1 | -1 | -1 | -1 | 2.71 |
| -1 | 1 | 1 | -1 | -1 | -1 | 3.48 |
| 1 | 1 | 1 | -1 | -1 | 1 | 3.79 |
| -1 | -1 | -1 | 1 | -1 | 1 | 2.17 |
| 1 | -1 | -1 | 1 | -1 | -1 | 2.10 |
| -1 | 1 | -1 | 1 | -1 | -1 | 2.51 |
| 1 | 1 | -1 | 1 | -1 | 1 | 2.82 |
| -1 | -1 | 1 | 1 | -1 | -1 | 2.30 |
| 1 | -1 | 1 | 1 | -1 | 1 | 2.50 |
| -1 | 1 | 1 | 1 | -1 | 1 | 3.12 |
| 1 | 1 | 1 | 1 | -1 | -1 | 3.13 |
| -1 | -1 | -1 | -1 | 1 | 1 | 2.46 |
| 1 | -1 | -1 | -1 | 1 | -1 | 2.61 |
| -1 | 1 | -1 | -1 | 1 | -1 | 3.12 |
| 1 | 1 | -1 | -1 | 1 | 1 | 3.20 |
| -1 | -1 | 1 | -1 | 1 | -1 | 2.62 |
| 1 | -1 | 1 | -1 | 1 | 1 | 2.71 |
| -1 | 1 | 1 | -1 | 1 | 1 | 3.39 |
| 1 | 1 | 1 | -1 | 1 | -1 | 3.52 |
| -1 | -1 | -1 | 1 | 1 | -1 | 2.18 |
| 1 | -1 | -1 | 1 | 1 | 1 | 2.51 |
| -1 | 1 | -1 | 1 | 1 | 1 | 2.83 |
| 1 | 1 | -1 | 1 | 1 | -1 | 2.90 |
| -1 | -1 | 1 | 1 | 1 | 1 | 2.29 |
| 1 | -1 | 1 | 1 | 1 | -1 | 2.46 |
| -1 | 1 | 1 | 1 | 1 | -1 | 3.09 |
| 1 | 1 | 1 | 1 | 1 | 1 | 3.19 |

^a^ Experiment II.

^b^ Values are expressed per gram of dry sugarcane bagasse added at the beginning.

**Table S3.** Solubilized phosphorus by *Aspergillus niger* FS1 in a solid-state fermentation as a function of combinations of different levels of biochar, rock phosphate (RP), sucrose, minerals, and moisture. The combinations were set up according to a 2^5-2^ fractional factorial design (¼ fraction) and the fermentations were performed for 7 days at 30 °C using sugarcane bagasse as substrate.

| **Experiment** | **Biochar** | **RP** | **Sucrose** | **Minerals^b^** | **Moisture** | **Solubilized P** |
| --- | --- | --- | --- | --- | --- | --- |
|  | **(mg g^-1^)^a^** | **(mg g^-1^)** | **(mg g^-1^)** | **(mL g^-1^)** | **(mL g^-1^)** | **(mg g^-1^)** |
| III | 80 (-1)^c^ | 35 (-1) | 35 (-1) | 1.00 (1) | 4 (1) | 2.68 |
|  | 120 (1) | 35 | 35 | 0.33 (-1) | 2 (-1) | 2.43 |
|  | 80 | 65 (1) | 35 | 0.33 | 4 | 2.72 |
|  | 120 | 65 | 35 | 1.00 | 2 | 2.65 |
|  | 80 | 35 | 65 (1) | 1.00 | 2 | 2.51 |
|  | 120 | 35 | 65 | 0.33 | 4 | 3.33 |
|  | 80 | 65 | 65 | 0.33 | 2 | 3.17 |
|  | 120 | 65 | 65 | 1.00 | 4 | 3.67 |
|  | 100 (0) | 50 (0) | 50 (0) | 0.67 (0) | 3 (0) | 3.13 |

^a^ Values are expressed per gram of dry sugarcane bagasse added at the beginning.

^b^ A solution of minerals was used (adapted from the Czapek’s medium) (g L^-1^): MgSO_4_.7H_2_O, 3; KCl, 3; FeCl_3_.6H_2_O, 0.108.

^c^ Values within the parentheses are the coded levels.

**Table S4.** Steepest ascent technique used to achieve a near-stationary region. The data used in the path calculation were obtained in the experiment III (Table S3). The changes in the factor levels were based on a 5- and 10-unit change in sucrose (highest “Unit x *f_t_*” value) in the experiments IV and V respectively.

|  | **Biochar**  **(mg g^-1^)^a^** | **RP**  **(mg g^-1^)** | **Sucrose**  **(mg g^-1^)** | **Minerals**  **(mL g^-1^)^b^** | **Moisture**  **(mL g^-1^)** | **Solubilized P^c^**  **(mg g^-1^)** |
| --- | --- | --- | --- | --- | --- | --- |
| Base level (0)^c^ | 100 | 50 | 50 | 0.67 | 3 |  |
| Unit^c^ | 20 | 15 | 15 | 0.33 | 1 |  |
| Estimated coefficient *f_t_*  (change in P per unit)^c^ | 0.125 | 0.159 | 0.276 | -0.018 | 0.205 |  |
| Unit x *f_t_* | 2.49 | 2.39 | 4.15 | -0.01 | 0.21 |  |
| Change in level per 5 change in sucrose^d^ | 3.008 | 2.882 | 5.00 | -0.007 | 0.247 |  |
| Path | 100 | 50.0 | 50.0 | 0.670 | 3.00 |  |
|  | 103 | 52.9 | 55.0 | 0.663 | 3.25 |  |
|  | 106 | 55.8 | 60.0 | 0.655 | 3.49 |  |
|  | 109 | 58.6 | 65.0 | 0.648 | 3.74 |  |
| Experiment IV^e^ | 112 | 61.5 | 70.0 | 0.641 | 3.99 | 3.5 b |
|  | 115 | 64.4 | 75.0 | 0.634 | 4.24 | 3.6 b |
|  | 118 | 67.3 | 80.0 | 0.626 | 4.48 | 3.9 ab |
|  | 121 | 70.2 | 85.0 | 0.619 | 4.73 | 4.1 a |
|  | 124 | 73.1 | 90.0 | 0.612 | 4.98 | 4.2 a |
| Change in level per 10 change in sucrose | 6.015 | 5.765 | 10.00 | -0.015 | 0.494 |  |
| Experiment V^e^ | 130 | 79 | 100 | 0.60 | 5.47 | 4.3 b |
|  | 136 | 85 | 110 | 0.57 | 5.97 | 4.8 ab |
|  | 142 | 90 | 120 | 0.57 | 6.46 | 5.1 ab |
|  | 148 | 96 | 130 | 0.53 | 6.96 | 5.1 ab |
|  | 154 | 102 | 140 | 0.53 | 7.45 | 4.9 ab |
|  | 160 | 108 | 150 | 0.53 | 7.94 | 5.1 ab |
|  | 166 | 113 | 160 | 0.50 | 8.44 | 5.3 a |
|  | 172 | 119 | 170 | 0.50 | 8.93 | 4.8 ab |

The calculations were done according Box and Wilson (1951).

^a^ Values are expressed per gram of dry sugarcane bagasse added at the beginning.

^b^ A solution of minerals was used (adapted from the Czapek’s medium) (g L^-1^): MgSO_4_.7H_2_O, 3; KCl, 3; FeCl_3_.6H_2_O, 0.108.

^c^ Obtained in the experiment shown in Table S3.

^d^ Based on glucose because of the highest value of “Unit x *f_t_*” for this factor.

^e^ For each experiment, means sharing a letter are not significantly different (Tukey test, *P* < 0.05).

**Table S5.** Solubilized phosphorus by *Aspergillus niger* FS1 in a solid-state fermentation as a function of combinations of different levels of biochar, rock phosphate (RP), sucrose, minerals, and moisture. Experiment VI was performed under a composite central design. Additional points were included in the design (experiments VII-IX) in order to improve the model adjustment. The fermentations were performed for 7 days at 30 °C using sugarcane bagasse as substrate.

| Experiment | Biochar | RP | Sucrose | Minerals | Moisture | Solubilized P (mg g^-1^)^a^ | |
| --- | --- | --- | --- | --- | --- | --- | --- |
|  | **(mg g^-1^)^a^** | **(mg g^-1^)** | **(mg g^-1^)** | **(mL g^-1^)^b^** | **(mL g^-1^)** | **Measured** | **Predicted^c^** |
| VI | 116 | 70 | 95 | 0.28 | 6.97 | 4.54 | 4.49 |
|  | 156 | 70 | 95 | 0.28 | 4.97 | 4.21 | 4.16 |
|  | 116 | 100 | 95 | 0.28 | 4.97 | 4.26 | 4.16 |
|  | 156 | 100 | 95 | 0.28 | 6.97 | 4.81 | 4.95 |
|  | 116 | 70 | 125 | 0.28 | 4.97 | 4.32 | 4.35 |
|  | 156 | 70 | 125 | 0.28 | 6.97 | 5.21 | 5.18 |
|  | 116 | 100 | 125 | 0.28 | 6.97 | 5.67 | 5.09 |
|  | 156 | 100 | 125 | 0.28 | 4.97 | 4.67 | 4.90 |
|  | 116 | 70 | 95 | 0.85 | 4.97 | 3.76 | 3.96 |
|  | 156 | 70 | 95 | 0.85 | 6.97 | 4.62 | 4.70 |
|  | 116 | 100 | 95 | 0.85 | 6.97 | 4.95 | 4.69 |
|  | 156 | 100 | 95 | 0.85 | 4.97 | 4.20 | 4.42 |
|  | 116 | 70 | 125 | 0.85 | 6.97 | 4.98 | 4.89 |
|  | 156 | 70 | 125 | 0.85 | 4.97 | 4.68 | 4.65 |
|  | 116 | 100 | 125 | 0.85 | 4.97 | 4.49 | 4.56 |
|  | 156 | 100 | 125 | 0.85 | 6.97 | 5.40 | 5.44 |
|  | 96 | 85 | 110 | 0.57 | 5.97 | 4.73 | 4.60 |
|  | 176 | 85 | 110 | 0.57 | 5.97 | 4.91 | 5.15 |
|  | 136 | 55 | 110 | 0.57 | 5.97 | 4.57 | 4.49 |
|  | 136 | 115 | 110 | 0.57 | 5.97 | 4.86 | 4.94 |
|  | 136 | 85 | 80 | 0.57 | 5.97 | 4.21 | 4.21 |
|  | 136 | 85 | 140 | 0.57 | 5.97 | 4.81 | 5.10 |
|  | 136 | 85 | 110 | 0 | 5.97 | 4.72 | 4.90 |
|  | 136 | 85 | 110 | 1.13 | 5.97 | 4.84 | 4.90 |
|  | 136 | 85 | 110 | 0.57 | 3.97 | 4.36 | 3.87 |
|  | 136 | 85 | 110 | 0.57 | 7.97 | 5.12 | 4.94 |
|  | 136 | 85 | 110 | 0.57 | 5.97 | 4.87 | 4.90 |
| VII | 300 | 90 | 140 | 0 | 7.5 | 6.42 | 6.45 |
|  | 350 | 90 | 140 | 0 | 7.5 | 6.74 | 6.68 |
|  | 400 | 90 | 140 | 0 | 7.5 | 6.80 | 6.85 |
|  | 450 | 90 | 140 | 0 | 7.5 | 6.76 | 6.97 |
|  | 500 | 90 | 140 | 0 | 7.5 | 7.25 | 7.02 |
|  | 250 | 90 | 155 | 0 | 7.5 | 6.16 | 6.19 |
|  | 250 | 90 | 170 | 0 | 7.5 | 5.85 | 6.11 |
|  | 250 | 90 | 185 | 0 | 7.5 | 5.80 | 5.91 |
|  | 250 | 90 | 200 | 0 | 7.5 | 5.80 | 5.58 |
| VIII | 500 | 90 | 190 | 0 | 7.5 | 7.30 | 7.59 |
|  | 600 | 90 | 190 | 0 | 7.5 | 8.13 | 7.88 |
|  | 700 | 90 | 190 | 0 | 7.5 | 8.32 | 7.92 |
|  | 800 | 90 | 190 | 0 | 7.5 | 7.66 | 7.71 |
|  | 900 | 90 | 190 | 0 | 7.5 | 7.16 | 7.26 |
|  | 1000 | 90 | 190 | 0 | 7.5 | 6.73 | 6.57 |
| IX | 1000 | 0 | 200 | 0 | 9 | 1.02 | 1.05 |
|  | 1000 | 25 | 200 | 0 | 9 | 2.93 | 2.88 |
|  | 1000 | 50 | 200 | 0 | 9 | 4.20 | 4.45 |
|  | 1000 | 150 | 200 | 0 | 9 | 8.43 | 8.19 |
|  | 1000 | 175 | 200 | 0 | 9 | 7.54 | 8.49 |
|  | 1000 | 200 | 200 | 0 | 9 | 9.06 | 8.53 |
|  | 150 | 115 | 110 | 0 | 10 | 4.11 | 4.11 |
| Confirmatory point | 850 | 175 | 200 | 0 | 7 | 7.59 | 9.27 |

^a^ Values are expressed per gram of dry sugarcane bagasse added at the beginning.

^b^ A solution of minerals was used (adapted from the Czapek’s medium) (g L^-1^): MgSO_4_.7H_2_O, 3; KCl, 3; FeCl_3_.6H_2_O, 0.108.

^c^ Calculated with the regression equation fitted:  = -7.73547 – 0.00132875 x_1_ + 0.0365891 x_2_ + 0.0643370 x_3_ + 1.75249 x_4_ – 0.0000122156 x_1_^2^ – 0.000204188 x_2_^2^ – 0.000270675 x_3_^2^ – 0.124354 x_4_^2^ + 0.0000416586 x_1_x_2_ + 0.0000729537 x_1_x_3_ (Y: predicted solubilized P, x_1_: level of biochar, x_2_: level of RP, x_3_: level of sucrose, x_4_: level of moisture)
